# The Big Five Personality Traits and Positive Orientation in Polish Adults with Multiple Sclerosis: The Role of Meaning in Life

**DOI:** 10.3390/ijerph19095426

**Published:** 2022-04-29

**Authors:** Małgorzata Szcześniak, Andrzej Potemkowski, Waldemar Brola, Zdzisław Kroplewski, Roman Ryszard Szałachowski, Marek Zak, Maciej Wilski, Piotr Sobolewski, Halina Bartosik-Psujek, Katarzyna Kapica-Topczewska, Joanna Tarasiuk, Agata Czarnowska, Alina Kułakowska, Beata Zakrzewska-Pniewska, Katarzyna Kubicka-Bączyk, Natalia Morawiec, Monika Adamczyk-Sowa, Adam Stępień, Jacek Zaborski, Anna Ratajczak, Marcin Ratajczak

**Affiliations:** 1 Institute of Psychology, University of Szczecin, 71-017 Szczecin, Poland; andrzej.potemkowski@wp.pl (A.P.); zdzislaw.kroplewski@usz.edu.pl (Z.K.); roman.szalachowski@usz.edu.pl (R.R.S.); 2 Collegium Medicum, Jan Kochanowski University, 25-317 Kielce, Poland; wbrola@wp.pl (W.B.); mkzak@ujk.edu.pl (M.Z.); piotrsobolewski@poczta.onet.pl (P.S.); 3 Department of Adapted Physical Activity, Poznan University of Physical Education, 61-871 Poznań, Poland; mwilski@wp.pl; 4 Department of Neurology, Institute of Medical Sciences, Medical College of Rzeszów University, 35-959 Rzeszów, Poland; bartosikpsujek@op.pl; 5 Department of Neurology, Medical University of Białystok, 15-276 Białystok, Poland; katarzyna-kapica@wp.pl (K.K.-T.); joanna.tarasiuk@umb.edu.pl (J.T.); agata.czarnowska@umb.edu.pl (A.C.); alina.kulakowska@umb.edu.pl (A.K.); 6 Department of Neurology, Medical University of Warsaw, 02-097 Warszawa, Poland; beata.zakrzewska-pniewska@wum.edu.pl; 7 Department of Neurology, Faculty of Medical Sciences in Zabrze, Medical University of Silesia, 40-055 Katowice, Poland; hib333@interia.pl (K.K.-B.); nataliamorawiec007@gmail.com (N.M.); msowa@sum.edu.pl (M.A.-S.); 8 Department of Neurology, Military Institute of Medicine, 01-755 Warszawa, Poland; astepien@wim.mil.pl; 9 Department of Neurology and Neurological Rehabilitation and Stroke Sub-Division, Specialist Hospital in Międzylesie, 04-749 Warszawa, Poland; jaczab@poczta.onet.pl; 10 Postgraduate Study, Pomeranian Medical University, 70-204 Szczecin, Poland; anna.ratajczak@euromedis.pl; 11 Clinical Trial Center for MS-Patients, 70-111 Szczecin, Poland; marcin.ratajczak@euromedis.pl

**Keywords:** big five traits, positive orientation, meaning in life, presence of life, searching for meaning, multiple sclerosis, adults

## Abstract

Scientific achievements concerning the direct relation between personality traits and positive orientation among patients with multiple sclerosis do not explain the role of potential mediators. In fact, some researchers argue that the traits–positivity association is much more complex than it seems to be. For this reason, we made an attempt to analyze the indirect relationship between the above-mentioned variables, including meaning in life as a mediator. In total, 618 patients with MS took part in the study. The NEO Five-Factor Inventory, the Positive Orientation Scale, and the Meaning in Life Questionnaire were used. The results showed that positive orientation/the presence of meaning/searching for meaning correlated positively with extraversion, openness to experience, agreeableness, and conscientiousness, and were negatively associated with neuroticism. Moreover, meaning in life in both its dimensions acted as a mediator in 9 of 10 models. It can be assumed that a propensity to establish interpersonal relationships (extraversion), use active imagination (openness), inspire confidence among others (agreeableness), and take responsibility (conscientiousness) can have an impact on someone’s positive attitude toward oneself and the surrounding world (positive orientation) when people have meaning in life and when they are seeking it.

## 1. Introduction

Multiple sclerosis (MS) is a chronic, progressive, inflammatory, and neurological disease [1,2,3,4] which is often accompanied by a large spectrum of clinical symptoms that include fatigue [5], sleep disturbance [5], pain or discomfort [4], social isolation [6], motor difficulties [7], cognitive impairment [8,9], hippocampal atrophy [10], and several others. According to the data available in the Atlas of MS [11] for 2020, there are 2.8 million people living with MS worldwide. The European prevalence of MS has recently risen to 143 per 100,000, being the highest among the continents. In Poland, the total number of people with MS is 46,000, with a prevalence of 120 per 100,000. These statistics indicate that Poland is one of the countries with a high frequency of MS [12,13].

Individuals diagnosed with MS often face the unpredictability of their illness [6,14,15] and challenges related to psychological, social, and economic functioning [6,7]. At the same time, the issue of well-being and its indicators (for example, personality traits) in this specific group of patients continues to be largely understudied [16,17,18]. However, although different dimensions of life quality in people with MS have been found to be considerably lower compared to the healthy population [19,20,21,22,23], there is an increasing body of empirical evidence that well-being co-occurs with ill-being [24]. For example, Dennison et al. [25], based on previous cross-sectional studies, identified correlates of better adjustment in people with MS: positive reappraisal, problem-focused strategies, seeking social support, family cohesion, self-efficacy, personal control, dispositional optimism, positive expectations of the future, positive affect, religiosity/spirituality, and hope. Recent research [26,27] has suggested that personality traits are not indifferent in the psychological well-being of people affected by MS and should be included in the analyses of their functioning. Optimism is also encompassed among different factors, indicating individual differences in this functioning [28]. Moreover, a few studies [29] examining sense-making in MS have displayed that redefined purpose of life is one of the strongest predictors of better adjustment to the disease. In fact, people with chronic disease conditions are exposed to hard situations on a daily basis which require from them some kind of reassessment of life [30].

Since the topic of the direct and indirect relationship between personality traits [27,31], meaning in life [29], and positive orientation is poorly understood and the research on the positive aspects in people with MS is still scarce [17,32], we chose two main goals for the present study. The first aim was to verify how the Big Five personality traits and meaning of life correlate with positive orientation. The second aim was to examine whether meaning of life mediates the relationship between personality traits and positive orientation.

### 1.1. Big Five and Positive Orientation

Personality has been identified as a key variable elucidating individual differences in various dimensions of well-being [31,33]. Personality is also considered an important constituent of clinical work with MS patients [34]. Regardless of the diverse attempts to define this concept, personality is commonly considered “as the relatively enduring patterns of thoughts, feelings, and behaviors that distinguish individuals from one another” [35] (p. 31).

Comprehensive research on the topic [14,36,37] shows that the Big Five model, consisting of phenotypic traits of extraversion, openness, conscientiousness, agreeableness, and neuroticism, is the most universally mentioned in the context of health and disease. According to different researchers, MS patients, compared with healthy controls, present lower levels of sociability and communication with others [32,38,39,40,41,42], deliberation, achievement, and persistence [31,43,44], cooperation and altruism [31,36], and desire for new knowledge and experiences [36]. At the same time, they have a proclivity for emotional reactions to problems, anxiety, and negative moods [31,36,39].

While the Big Five traits address behavioral dispositions [45], positive orientation, conceptualized “as a pervasive mode of appraising, viewing, and construing that significantly affects how individuals predispose themselves to actions and experiences” [46] (p. 77), represents a salient attitude that is essential for facing serious challenges such as illness [45]. Over the years, correlational findings among the healthy population have revealed that positive orientation was linked positively with extraversion, agreeableness, openness to experience, and conscientiousness, and negatively with neuroticism [46]. Likewise, Przepiorka et al. [47] obtained similar results among participants from Chile, Hong Kong, and Poland. Moreover, Szcześniak et al. [48] reported comparable outcomes, except for openness to experience, which was not associated significantly with positive orientation. Based on previous studies, we assumed that:

**Hypothesis** **H1** **(H1).** *Positive orientation positively correlates with extraversion, openness to experience, agreeableness, and conscientiousness, and is negatively associated with neuroticism.*

### 1.2. Big Five and Meaning in Life

MS, like other illness-related conditions, requires a continual process of adaptation to unexpected hardships [29]. One promising psychological phenomenon which is considered to help in overcoming difficulties related to chronic discomfort is meaning in life [49], which has been called “an antidote to the unknown” [50] (p. 3).

Although there are different theories of what meaning in life is [51], researchers have developed definitions that share three common facets: coherence, purpose, and significance [52,53]. In the present study, we adopted Steger’s conceptualization of meaning in life that refers to people’s desire to comprehend their experience and identify goals to achieve in the future [51].

People differ in the degree to which they are committed to have and actively search for meaning [54,55]. Therefore, meaning in life is understood both as the presence of and searching for meaning [56]. Individuals have meaning in life when they feel that their life is relevant [49] and comprehend themselves and the world around them [54]. Instead, searching for meaning consists of the drive to discover meaning in one’s life [57]. This distinction is important, because depending on whether we are dealing with the presence or the search for meaning, the relationship between both dimensions and the personality may be different. For example, outcomes obtained by Steger et al. [54] show that people declaring the presence of meaning in life tend to experience lower psychological distress and negative emotions (neuroticism), higher trust and altruism (agreeableness), greater joy in social interactions (extraversion), and competence and proactivity (conscientiousness). Conversely, people who are searching for meaning express higher levels of anxiety (neuroticism) and curiosity and imagination (openness to experience). In another study, Schnell and Becker [58] found positive correlations between meaningfulness and extraversion, openness, conscientiousness, and agreeableness, and the lack of an association with neuroticism. Moreover, the four significantly correlating personality traits acted as predictors of meaningfulness, explaining 16% of the variance. Considering prior research, we assumed that:

**Hypothesis** **H2** **(H2).** *Meaning in life correlates positively with extraversion, openness to experience, agreeableness, and conscientiousness, and is negatively associated with neuroticism.*

### 1.3. Meaning in Life and Positive Orientation

A good starting point for understanding the relationship between meaning in life and positive orientation is to look at the conceptual structure of the latter construct. According to Caprara et al. [59], positive orientation is a common high-order factor that explains self-esteem, life satisfaction, and optimism. Since presence and searching for meaning correlate with self-esteem [60,61,62], life satisfaction [60,63,64,65,66,67], and optimism 60,64,65,67], it can be assumed that meaning in life may be associated with positive orientation.

Moreover, Oleś and Jankowski [68], based on theoretical and empirical research, advanced the extended model of positive orientation that besides its three basic key facets also includes purpose in life. This is because when people view themselves, their lives, and their future confidently and optimistically, they are also more inclined to master life challenges, regardless of defeats or suffering. Hence, positive orientation can be perceived as an important cognitive disposition to consider life circumstances as meaningful and integral to one’s own experience [69]. Other studies have shown that people who present higher meaning in life report a more positive health orientation [70]. Chan et al. [71] found that meaning in life coexists with positive orientation among helping professionals working in settings where they are confronted with pain and death.

Park et al. [72] observed that people with greater meaning, both the presence of and the search for, declare higher levels of life satisfaction and happiness. Several researchers consider meaning in life as a key variable in the framework of positive functioning in different stages of development [73]. Since life satisfaction refers to people’s positive evaluation of their life, and happiness also reflects, among other aspects, positivity [68], it can be assumed that meaning in life may be a predictor of positive orientation. In fact, longitudinal evidence has confirmed that meaning is an antecedent of life satisfaction [63], a source of well-being [72,74], a predictor of self-esteem [75] and interpersonal well-being outcomes [76], and a contributor to personal growth [77]. Taking into account the previous findings, we suggested that:

**Hypothesis** **H3** **(H3).** *Meaning in life (presence and searching) correlates positively with positive orientation.*

### 1.4. Meaning in Life as a Mediator

The scientific achievements presented so far concerning the direct relation between personality traits and positive orientation do not explain the role of potential mediators. In fact, some researchers argue that the traits–positivity association is much more complex than it seems to be [78]. Likewise, Strickhouser and colleagues [79], based on a meta-analysis of 47 studies, underline that more work is required to explore the mediational mechanisms and moderators that explain the relationship between personality and health-related outcomes. For this reason, we made an attempt to analyze the indirect relationship between the above-mentioned variables, including meaning in life as a mediator. The rationale behind this choice stands in the fact that meaning in life has been acknowledged as an essential aspect of the human motivational system [80] and a mediator between different personality dimensions and psychological well-being [81]. Moreover, meaning in life was predicted by the Big Five traits in different studies [55] and it was itself a predictor of well-being [76,82], quality of life [82], and optimism [83]. Considering the theoretical premises and empirical research, we hypothesized that:

**Hypothesis** **H4** **(H4).** *Meaning in life is a mediator between personality traits and positive orientation.*

### 1.5. Confounding Hypothesis

After assuming direct and mediation associations between the Big Five factors, positive orientation, and meaning in life, we also addressed the issue of potential confounders that could falsely alter the relationships [84] between personality traits and positivity. Based on the empirical literature, we presumed that the following extraneous variables could reduce or increase these relationships: age, sex, educational background, disease duration, clinical severity/course of the disease, ways of getting around with the disease, employment, and financial assistance. The rationale behind choosing the above listed possible confounders stems from the research which indicates that there are some differences in the Big Five traits and age [85,86], sex [87,88,89], educational background [90,91], disease duration [92], disease severity [93] or progressive disease course [36], employment [94], and financial assistance [95]. Likewise, a similar pattern of relationships concerns the association between positive orientation and age [96], sex [97], educational background [98,99], disease duration [7], disease severity [100] or progressive disease course [101], employment [102], and financial assistance [103]. Following the empirical evidence from previous studies, we assumed that:

**Hypothesis** **H5** **(H5).** *Age, sex, educational background, disease duration, disease severity or progressive disease course, employment, and financial assistance may confound the relationship between personality traits and positive orientation.*

## 2. Materials and Methods

### 2.1. Ethics Approval

This project research was authorized by the Bioethics Committee of the Institute of Psychology at the University of Szczecin (KB 15/2019) and was performed in accordance with the criteria of the Declaration of Helsinki.

### 2.2. Participants

In total, 618 patients with MS took part in the study. Their mean age was *M* = 42.26 (*SD* = 11.40; range = 17–70). Most respondents were women (*n* = 446; 72%). With respect to the respondents’ educational background, 3% declared a primary education, 15% a vocational school education, 35% a high school education, and 47% a higher education. In terms of years of disease duration (since the official date of diagnosis), the average was *M* = 9.12 (*SD* = 7.27). With respect to disease severity/course, approximately 41% of patients had relapsing and remitting MS, 38% did not have relapses, and 18% experienced slowly progressive deterioration. Only 3% did not reply to this question. Most of the patients responded that they moved without the help of devices and third persons (79.3%). Other people used the help of devices or third parties. About 55% of respondents worked full-time, 28% had a disability pension, and 10% worked part-time. The others either did not work or gave no answer. Regarding the issue of financial support, the majority declared that they did not receive any aid (55%).

Patients with MS represented the following clinical centers where they remain under the medical care of neurologists focusing on the treatment of this demyelinating disease: Białystok, Końskie, Międzylesie, Rzeszów, Sandomierz, Szczecin, Warszawa, and Zabrze. Qualification criteria considered: (1) age from 18 to 65 (despite this criterion, a few patients younger than 18 and older than 65 who wanted to participate in the study were included); (2) the “McDonald criteria” 2010/2017; (3) informed consent to be involved in the research. Exclusion criteria comprised: (1) advanced medical condition that prevents a person from taking part in the study, such as: cognitive or speech impairment; (2) the coexistence of neoplastic diseases. All participants provided written informed consent prior to the study. The adolescents under 18 took part in the research after acquiring their parents’ or legal guardians’ approval.

### 2.3. NEO Five-Factor Inventory (NEO-FFI)

The NEO-FFI is a self-report inventory, developed by Costa and McCrae [104] and adapted into Polish by Zawadzki et al. [105], which measures five personality domains: neuroticism (e.g., anxiety, insecurity, and maladjustment), extraversion (e.g., sociability, playfulness, and assertiveness), openness to experience (e.g., originality, creativity, and imagination), agreeableness (e.g., trust, empathy, and cooperation), and conscientiousness (e.g., carefulness, responsibility, and order). This version of the Big Five inventories is extensively used in clinical settings among patients with MS [34,106]. The questionnaire consists of 60 items which are assessed on a five-point Likert scale (1 = *strongly disagree* and 5 = *strongly agree*). The NEO-FFI is a well-validated measure. In the present study, five factors presented very good internal consistency: neuroticism (α = 0.89), extraversion (α = 0.83), openness to experience (α = 0.77), agreeableness (α = 0.88), and conscientiousness (α = 0.91). The overall Cronbach’s alpha was excellent (α = 0.94).

### 2.4. Positive Orientation Scale (P-Scale)

The P-Scale [46,107], in terms of the Polish adaptation by Łaguna et al. [108], is a single-factor measure developed to assess positive orientation considered as a higher-order latent variable that reflects self-esteem (e.g., “I feel I have many things to be proud of”), life satisfaction (e.g., “On the whole, I am satisfied with my life”), and optimism (e.g., “I look forward to the future with hope and enthusiasm”). The scale is composed of 8 items and has a one-factor structure. In the current study, the one-factor structure was confirmed and explained 62.41% of the variance. Participants rated their positivity level using a 5-point scale ranging from 1 = *strongly disagree* to 5 = *strongly agree.* Item 4 is negatively worded (e.g., “At times, the future seems unclear to me”). The psychometric properties of the P-Scale were very good. The overall Cronbach’s alpha was equal α = 0.90.

### 2.5. Meaning in Life Questionnaire (MLQ)

The MLQ authored by Steger et al. [60] and adapted into Polish by Kossakowska et al. [109] consists of two 5-item subscales. The first subscale assesses the presence of meaning in life (e.g., “My life has a clear sense of purpose”). The second subscale measures the search for meaning in life (e.g., “I am looking for something that makes my life feel meaningful”). In the present study, the two-factor structure was confirmed and explained 73.14% of the variance (presence—41.18% and searching—31.96%). The respondents assessed their subjective sense of and drive for meaning on a 7-point Likert scale, ranging from 1 = *absolutely untrue* to 7 = *absolutely true*. Item 9 is negatively worded and needs to be reversed (e.g., “My life has no clear purpose”). Both subscales have good internal consistency. In the present study, the subscale of presence of meaning (α = 0.88) and the subscale of searching for meaning showed very good coefficients (α = 0.88).

### 2.6. Data Analysis

SPSS version 20 was used to explore the descriptive and correlational statistics for the Big Five traits, positive orientation, and both dimensions of meaning in life. We implemented the criterion of values for skewness and kurtosis between ±2 as recognized for relatively normal distributed variables. Since the data were not normally distributed, the Spearman correlation was run to calculate the relationship between personality traits, positive orientation, and the presence of and searching for meaning.

The G*Power 3.1.9.4 software [110] was used to select appropriate sample size values [111]. In an a priori analysis, we considered the small effect size (*r* = 0.15), the maximum value of the α = 0.01 coefficient, and the recommended power equal to 0.85. The analysis showed the minimum size at the level of 574 respondents. The rationale for selecting the small effect for this research design is based on empirical evidence which presents inconsistent correlations between the Big Five factors and positive orientation, from none [112,113], through low [112,113], to medium [48,113].

A linear regression model was used to: check for the occurrence of multicollinearity, identify outliers, and adjust for potential confounders. In order to detect multicollinearity, the Variance Inflation Factor (VIF) and tolerance values were applied [114]. Following the general rule, it was assumed that the VIF values should not exceed 5.0 [115] and tolerance values should not be less than 0.1 [116]. The Mahalanobis distance with the criterion of *p* < 0.001 and Cook’s distance with case values lower than 1 were implemented to verify the presence of outliers. According to the hypothesis (H5), age, sex, educational background, disease duration, clinical severity/course of the disease, ways of getting around with the disease, employment, and financial assistance were included in the model as potential confounders. A 10% cutoff was used as a commonly mentioned indicator of a confounding effect [117].

The significance of indirect effects was tested using the PROCESS macro (version 3.2) and Model 4, with 5000 bootstrap statistics.

## 3. Results

### 3.1. Preliminary Analyses

The Big Five personality traits, positive orientation, and the presence of and searching for meaning in life were analyzed to check the normality of their distribution. Since most factors exceeded the values of ±2, especially for kurtosis, we assumed that all of them were not normally distributed (Table 1).

### 3.2. Correlations

The outcomes of the Spearman correlations (Table 2) showed significant relationships between all the factors, mostly at the level of *p* < 0.001. More specifically, positive orientation correlated positively with extraversion, openness to experience, agreeableness, and conscientiousness, and was negatively associated with neuroticism, thus confirming hypothesis H1. Meaning in life in both dimensions showed a similar pattern of results. In fact, both the presence of and searching for meaning correlated positively with extraversion, openness to experience, agreeableness, and conscientiousness, and were inversely associated with neuroticism.

On the bases of the correlational findings, it can be deduced that patients with MS who are positively inclined to think and act, regard their lives as meaningful, or are motivated to find meaning are also more sociable, creative, empathetic, responsible, and less anxious.

### 3.3. Multicollinearity and Confounding Variables

The multiple linear regression outcomes showed that there was no problem with collinearity for the sample’s data since the range of VIF values was between 1.030 and 2.783, and the tolerance values were between 0.359 and 0.971. The Mahalanobis distance indicated the presence of 24 outliers. However, since the statistics with and without the abnormal data presented similar outcomes, we decided to not remove them. Moreover, Cook’s values were well below 1, with the range between 0.000 and 0.130. With respect to confounders (H5), the results showed that the variables included in Step 1 were not relevant. They accounted for only 4.7% of the variance (*R*^2^ = 0.047): age (*β* = −0.056; *t* = −1.725; *p* = 0.085), sex (*β* = 0.016; *t* = 0.570; *p* = 0.569), disease duration (*β* = −0.024; *t* = −0.777; *p* = 0.438), educational background (*β* = 0.021; *t* = 0.731; *p* = 0.465), clinical severity/course of the disease (*β* = −0.016; *t* = −0.550; *p* = 0.582), ways of getting around with the disease (*β* = 0.009; *t* = 0.285; *p* = 0.776), employment (*β* = −0.022; *t* = −0.740; *p* = 0.460), and financial assistance (*β* = 0.012; *t* = 0.424; *p* = 0.672). The Big Five traits and both dimensions of meaning in life explained an additional 48% of the variance.

### 3.4. Mediational Models

The mediation analysis presented in Table 3 showed that meaning in life in both its dimensions acted as a mediator in 9 of 10 models. In all nine cases, 95% bootstrap confidence intervals did not comprise zero.

## 4. Discussion

The aim of this research was to verify the existence of correlations between the studied variables and the role of the meaning in life as a mediator between personality traits and positive orientation. All of the hypotheses were fully confirmed, except for the lack of a mediating effect of searching for meaning between neuroticism and positivity.

Starting with the first hypothesis (H1), positive orientation was positively associated with extraversion, openness to experience, agreeableness, and conscientiousness, and inversely linked to neuroticism. The results are consistent with prior studies. For example, Zhang and Tsingan [72], based on research carried out among Chinese university students, found that the Big Five dimensions correlated in a similar way with the experience of positive affective states. Likewise, Ebert et al. [28] noticed that the extraversion, openness to experience, agreeableness, and conscientiousness of undergraduate psychology students were associated positively with dispositional optimism, which expresses a positive outlook toward life circumstances, while neuroticism correlated negatively. Such results may be due to the fact that the Big Five factors have been found to be crucial traits in determining people’s overall adaptation to stress related to disease and traumatic events [118] or to daily life. Personality is also considered to be a decisive determinant of well-being [79], whether we are dealing with people suffering from a disability and chronic disease, or those without comorbidities.

As for the hypothesis concerning the relationship between personality traits and meaning in life (H2), it also found its confirmation with the previous studies. For example, Strober [14] noticed that purpose of life, understood as a person’s feelings and goals concerning their life’s meaning, correlated positively with extraversion (*r* = 0.58 **), conscientiousness (*r* = 0.54 **), agreeableness (*r* = 0.33 **), and openness (*r* = 0.31 **), and negatively with neuroticism (*r* = −0.64 **). It should be noted that the results obtained in our study are consistent, both in terms of direction and strength, with the correlations between personality traits and meaning in life found by Strober in a study of patients with clinically defined MS. In a Turkish study of healthy participants, Işık and Üzbe [119] obtained small and moderate correlations between the Big Five dimensions and the presence of/searching for meaning in life. Additionally, their regressional results showed that openness to experience and neuroticism were, respectively, positive and negative predictors of the presence of meaning in life. Such outcomes suggest that people who are open-minded and creative, being at the same time less anxious, are more likely to understand their life experience and achieve their goals. Therefore, it can be concluded that personality traits are significant in the context of meaning in life.

The third hypothesis (H3) concerning the relationship between both dimensions of meaning in life and positive orientation was also supported. Due to the fact that there is no research on the relationship between the two variables, a good solution for understanding the obtained results is to refer to previous studies linking meaning in life with self-esteem, optimism, and life satisfaction, to which positive orientation alludes. For instance, Zhang et al. [61] found in a group of older adults between 65 and 73 that both the presence of and search for meaning correlated positively with self-esteem. Such an outcome suggests that people who have already recognized their unique meaning in life or are still looking for it feel valuable and have an overall better assessment of the self. Thus, meaning in life may contribute to the enhancement of self-esteem. Moreover, Barnett et al. [120] observed among hospice nurses that the presence of meaning in life promoted their self-esteem. This is in line with terror management theory, which suggests that the acceptance of inevitable aversive life conditions requires the need for their reorganization through a meaningful worldview which, in turn, leads to positive outcomes. Previous studies [121,122,123], regardless of the composition of the study groups (e.g., adolescents, university students, and the elderly), also indicate that overall meaning in life correlates with life satisfaction and the positive expectations which people hold for the time ahead [122]. An important aspect of the correlations obtained in the current study concerns the positive relationship between the search for meaning and positive orientation. In some previous studies, this association was negative. This means that, as Steger et al. [54] maintain, the concept of searching for meaning can be understood as a positive part of human life and not only as a sign of dysfunction. Since our research was conducted among patients with MS, it can be assumed that they consider looking for meaning as a typical and positive part of their life. Undoubtedly, it is difficult to think that people with MS have found meaning in life if they do not know what the disease and the immediate future will bring. The unpredictability of their condition makes chronically ill people look for meaning in life over and over again. Moreover, some researchers have noticed that the search for meaning is more valuable for people in difficult circumstances, such as disease, to the presence of meaning [123]. For example, searching for meaning was higher than the presence of meaning among adults with physical disabilities [124], high health anxiety [57], and chronically ill patients who, based on cluster analysis, showed the features of a poorly adapted group [73].

With respect to hypothesis H4, the presence of meaning and searching for meaning acted as mediators in the relationship between personality traits and positive orientation. On the basis of the obtained results, it can be carefully assumed that having a clear sense of purpose or looking for something that makes life feel meaningful may modify the direct association between the Big Five dimensions and the positive evaluation of different domains of people’s lives. To be explicit, it can be assumed that a propensity to establish interpersonal relationships, use active imagination, inspire confidence among others, and take responsibility can have an impact on someone’s positive attitude toward oneself and the surrounding world when people have meaning in life and when they are seeking it. The mediating nature of both dimensions of meaning in life seems to be of particular importance for people with chronic diseases, including those diagnosed with MS. These results are in line with some previous studies [29] which have shown that the struggle to find an explanation for one’s own conditions helps to reinstate meaning. Patients with MS who redefine their life purpose tend to report better adjustment to the disease through higher life satisfaction, positive states of mind, and lower anxiety and depression.

Hypothesis H5 was not confirmed. None of the potential confounders turned out to be a variable that would alter the direct relationship between the Big Five personality factors and positive orientation. Thanks to the lack of a confounding effect, we can cautiously assume that the selected variables did not obscure the association between personality traits and positivity. However, it cannot be excluded that these variables in a different research setting, cultural context, or in the presence of other variables may act as confounders.

## 5. Limitations

Although the current research was conducted on a large group of patients with MS, concerned psychological aspects of their functioning that are rarely studied, and included quite a large number of potential confounders, it also has its limitations. The first one is the cross-sectional nature of the study which prevented cause-and-effect conclusions. Since data are gathered at one specific point in time in cross-sectional research designs, the issue of the change process, characteristic of longitudinal studies, must rely on robust theory [125] or empirical research that justifies the use of an appropriate temporal sequence of variables. Therefore, in the future, it would be a good solution to use the longitudinal approach which could allow such conclusions to be drawn. The second limitation relates to the use of quantitative self-reported measures. Undoubtedly, the qualitative approach could also be used in future analyses, especially in the context of the presence of and search for meaning in life. Thirdly, although we adjusted the model for a number of potential confounders, there may be several other essential covariates, not included in the analysis, that could have a confounding effect on the relationship of interest (e.g., physical activity, psychological stress, the use of alcohol or other recreational drugs, smoking status, and the presence of comorbidities).

## 6. Conclusions and Implications

The outcomes of the current study may increase our understanding of meaning in life and its mediatory role in the direct relationship between personality traits and positive orientation given that there is relatively little research on the subject in the context of multiple sclerosis and chronic disease [126]. Based on our results, it can be cautiously assumed that a propensity to establish interpersonal relationships (extraversion), use active imagination (openness), inspire confidence among others (agreeableness), and take responsibility (conscientiousness) can have an impact on someone’s positive attitude toward oneself and the surrounding world when people have meaning in life and when they are seeking it.

Additionally, our findings may also have some implications in terms of therapeutic work with MS patients or other similar populations. More specifically, concern for the presence of and search for meaning in situations new to the course of the disease is not indifferent to the adaptive functioning of people suffering from MS or disability. In fact, it seems especially important to include the process of “meaning restoration” [126] as any chronic disease often threatens a person’s sense of meaning when challenged with serious health adversity. Therefore, the inclusion of non-pharmacological interventions, for example, using logotherapy or other forms of counseling, could be a helpful solution in the treatment of patients with MS, thus broadening their life perspective, opening them up to new opportunities, and contributing to their personal growth and psychosocial functioning.

## Figures and Tables

**Table 1:** Descriptive statistics for the NEO-FFI, P-Scale, and MLQ (*n* = 618).

Factors	*M*	*SD*	Skewness	Kurtosis
Neuroticism	35.03	9.89	−0.781	1.874
Extraversion	37.73	8.67	−1.451	6.816
Openness to experience	35.50	7.66	−1.844	8.381
Agreeableness	39.70	8.65	−2.057	7.407
Conscientiousness	42.73	9.37	−2.062	7.351
P-Scale	28.24	6.59	−1.864	5.825
Presence of meaning	22.99	6.38	−1.161	2.714
Searching for meaning	23.56	6.16	−1.390	3.728

**Table 2:** Spearman correlation coefficients between NEO-FFI, P-Scale, and MLQ (*n* = 618).

Factors	NE	EX	OP	AG	CO	P-S	PM	SFM
NE	1							
EX	−0.292 ***	1						
OP	−0.082 *	0.242 ***	1					
AG	−0.297 ***	0.321 ***	0.266 ***	1				
CO	−0.258 ***	0.508 ***	0.202 ***	0.474 ***	1			
P-S	−0.436 ***	0.502 ***	0.201 ***	0.360 ***	0.477 ***	1		
PM	−0.366 ***	0.446 ***	0.170 ***	0.300 ***	0.479 ***	0.678 ***	1	
SFM	−0.084 *	0.372 ***	0.206 ***	0.173 ***	0.334 ***	0.466 ***	0.535 ***	1

*** *p* < 0.001; * *p* < 0.05; NE—neuroticism; EX—extraversion; OP—openness to experience; AG—agreeableness; CO—conscientiousness; P-S—Positivity Scale; PM—presence of meaning; SFM—search for meaning.

**Table 3:** Mediational models of NEO-FFI, P-Scale, and MLQ (*n* = 618).

Models	a Path	b Path	c Path	c’ Path	Indirect Effect and B (SE)	95% CI LOWER UPPER
NE−PM−P-S	−0.09 ***	0.68 ***	−0.09 ***	−0.03 (ns)	(−0.0626; 0.0301)	(−0.1193; −0.0015)
NE−SFM−P-S	0.05 *	0.57 ***	−0.09 ***	−0.13 ***	(0.0331; 0.0254)	(−0.0118; 0.0870)
EX−PM−P-S	0.33 ***	0.57 ***	0.37 ***	0.17 ***	(0.1948; 0.0301)	(0.1377; 0.2566)
EX−SFM−P-S	0.29 ***	0.40 ***	0.37 ***	0.25 ***	(0.1217; 0.0265)	(0.0720; 0.1764)
OP−PM−P-S	0.25 ***	0.64 ***	0.28 ***	0.12 ***	(0.1637; 0.0358)	(0.0958; 0.2335)
OP−SFM−P-S	0.26 ***	0.49 ***	0.28 ***	0.15 ***	(0.1319; 0.0332)	(0.0694; 0.1994)
AG−PM−P-S	0.27 ***	0.61 ***	0.32 ***	0.14 ***	(0.1711; 0.0302)	(0.1131; 0.2303)
AG−SFM−P-S	0.21 ***	0.46 ***	0.32 ***	0.22 ***	(0.0996; 0.0269)	(0.0491; 01548)
CO−PM−P-S	0.31 ***	0.58 ***	0.33 ***	0.14 ***	(0.1868; 0.0282)	(0.1340; 0.2450)
CO−SFM−P-S	0.26 ***	0.42 ***	0.33 ***	0.22 ***	(0.1114; 0.0249)	(0.0659; 0.1629)

*** *p* < 0.001; * *p* < 0.05; ns—not significant; NE—neuroticism; EX—extraversion; OP—openness to experience; AG—agreeableness; CO—conscientiousness; PM—presence of meaning; SFM—search for meaning; a path—effect of Big Five factors on mediators (presence of and search for meaning); b path—effect of mediators on positive orientation; c path—effect of Big Five factors on positive orientation; c’ path—direct effect of Big Five factors on positive orientation while controlling for presence of and search for meaning.

## Data Availability

The data that support the findings of this study are available from the corresponding author, M.S., upon reasonable request.
